# Does Judo Training Contribute to the Motor Development of Children and Adolescents? A Systematic Review

**DOI:** 10.3390/jcm14072439

**Published:** 2025-04-03

**Authors:** Monika Kowalczyk, Małgorzata Zgorzalewicz-Stachowiak, Wiesław Błach, Maciej Kostrzewa

**Affiliations:** 1 Department of Health Prophylaxis, University of Medical Sciences, 61-701 Poznan, Poland; 2 Department of Physical Education & Sport, Wroclaw University of Sport and Health Sciences, 51-612 Wroclaw, Poland; 3 Institute of Sport Science, The Jerzy Kukuczka Academy of Physical Education in Katowice, 40-065 Katowice, Poland

**Keywords:** judo, physical activity, motor competence, children, adolescent

## Abstract

**Background**: Physical activity, including its organized form, is of key importance in the motor development of children and adolescents. Among the increasingly popular physical activities in this age group are martial arts, particularly judo. In addition to its educational and social values, this discipline, in its assumptions, gives practitioners the opportunity for motor development. This study aims to assess the development of motor competence in children and adolescents up to 15 years of age practicing judo. **Methods**: The study was conducted using seven databases (Scopus, Web of Science, PubMed, Embase, OVID, CINHAL Ultimate and SPOLIT) across 15 years (from January 2009 to May 2024). The PRISMA methodology was used to include studies, and the CASP protocol was applied to assess the quality of these studies. Inclusion criteria were developed based on PICOS. Finally, 22 studies that met the established criteria were included. **Results**: Most of the studies (21) were observational, and only one was a randomized controlled trial. The review indicated that regular judo training significantly improved muscle strength, endurance, speed, coordination, flexibility, balance and body posture compared to control groups. In contrast to some other sports, judo demonstrated comparable benefits in the development of motor abilities. **Conclusions**: Judo is a sport discipline that significantly affects the motor development of children and adolescents. Furthermore, regular participation in training helps to achieve the daily dose of moderate-to-vigorous activity recommended by the World Health Organization in this age group.

## 1. Introduction

Motor competence (MC) is the ability to perform a variety of motor acts including fine and gross motor skills, which have a key impact on daily functioning. They can be divided into locomotor skills (e.g., running, jumping and hopping), object control skills (e.g., catching and throwing) and stability skills (balance and postural control) [1,2,3,4]. Childhood is considered crucial for the development of these competencies [2,5,6]. Engaging children in physical activity (PA) increases their level of physical fitness, shapes positive habits and increases the chance of continuing it in later stages of life [6]. Moreover, acquiring simple motor skills in early childhood makes it possible to perform more complex movements, which encourages participation in more demanding physical activities and organized sports. Children with lower levels of MC may encounter difficulties in mastering sport-specific movements in the future, especially in practicing competitive sports [1]. The level of PA recommended by the World Health Organization (WHO) for children and adolescents aged 6–17 is related to physical and mental health, including physical fitness [7]. There is a strong relationship between MC and physical fitness in this age group, and these two factors even mutually complement one another [8,9]. Physical fitness consists of motor skills defined as directly related to health: health-related fitness (endurance, strength and flexibility) and skill-related fitness (including speed, agility, balance, coordination and power) [10,11]. Thus, MC is considered an important factor in shaping and maintaining health in children and adolescents.

PA plays a vital role in the development of all MCs. Unfortunately, interest in physical exercise and sports among children and adolescents is currently decreasing, while the trend of a sedentary lifestyle and increased use of electronic devices in free time is simultaneously increasing [10,12]. Children who are inactive in their youth usually remain inactive later in life [12]. Moreover, studies show that children with lower levels of PA have lower levels of physical fitness and motor skills, which can lead to negative health effects [2,3,13]. Lower involvement in PA is associated with the lower activation of the musculoskeletal system, which may increase the risk of developing cardiometabolic diseases, obesity, mental illness and osteoporosis in the future [14].

MC develops under the influence of biological factors that change with age as the body grows, but the most important factor remains the environmental influence assessed through involvement in PA, and primarily its form and time devoted to it [3,10,15]. From the youngest age, children should participate in indoor activities, free physical play at home and outdoor activities on public playgrounds and sports fields. Participating in organized sports activities creates broader development opportunities, where a child not only moves more but also develops the quality of these movements [6,16]. Martial arts are among the organized sports activities that are becoming increasingly popular [17]. They offer the possibility of physical development and have educational and social value. In recent years, judo has gained significant popularity around the world as a martial art and currently also as an Olympic sport [18]. Judo is practiced by millions of people around the world as a sport and as a recreational and health-promoting activity [19,20]. Kowalczyk et al. [19] pointed out that judo is practiced by children from the youngest age; that is, from the age of four. The education stage lasts until about 15 years of age and, at that time, judo training focuses on learning falls, throws, holds and fights in the form of randori and shai, which improve technical skills [20]. As new skills are acquired, practitioners achieve subsequent training levels (kyu), up to the master’s level of dan (black belt) [21]. This period is characterized by comprehensive physical development, which is combined with natural biological development and learning techniques and the philosophy of judo [19,20]. At the age of about 15–16—the cadet category U18 (under 18 years old) according to the International Judo Federation (IJF)—young people who decide to continue judo training enter a cycle of increased training load and competitions, which lasts through junior to senior age [20,22]. Professional judokas, striving to achieve the highest sporting goals, are expected to have high technical and tactical skills, as well as the appropriate physical and psychological preparation [23,24].

The latest published review concerned the analysis of the development of motor skills in children and adolescents practicing various martial arts [17]. The review included 16 studies of different martial arts interventions, but only four were devoted specifically to judo practiced by children aged 4–18 years. Another review focused on the broader aspects of the effectiveness of judo training, such as the physical, fitness-related, social and psychological, in children aged 4–12 years, but included only nine analyzed publications [25]. The review aimed to analyze the benefits of judo training for the development of MC in children and adolescents up to 15 years of age. In addition, the review was intended to investigate the level of MC achieved by participants in organized judo classes compared to the practitioners of other recreational sports and those who participate only in school physical education (PE). This information may be useful for PE teachers and judo coaches, as well as for parents and other groups promoting PA in children and adolescents.

## 2. Materials and Methods

### 2.1. Search Strategy

A systematic review was conducted using the Preferred Reporting Items for Systematic Reviews (PRISMA 2020) [26]. A systematic search was conducted in June 2024 in seven databases (Scopus, Web of Science, PubMed, Embase, OVID, CINHAL Ultimate and SPOLIT). The search covered the last 15 years (from January 2009 to May 2024). Only peer-reviewed journals written in English were considered for eligibility. Search terms were identified with Medical Subject Headings (MeSH). Records were searched using various combinations of the following search terms: judo AND (children OR boy OR girl OR adolescent OR youth OR teen) AND (motor skill OR motor competence OR motor performance OR posture OR balance OR strength OR speed OR endurance OR flexibility OR coordination).

### 2.2. Eligibility Criteria

Studies were included if they met the following PICOS (Population, Intervention, Comparison, Outcomes and Study) criteria: healthy children and adolescents up to 15 years at baseline (mean age) without any declared diseases (Population); involved in judo training (Intervention); included inactive control group or/and active comparator, except for judo (Comparison); examined motor skills, but not limited to any specific variables (Outcomes), and observational (cohort, cross-sectional) or interventional (pre-post study design)—both nonrandomized and randomized (Study design) [27].

### 2.3. Data Extraction

One author, Monika Kowalczyk, carried out the initial search and two authors, Monika Kowalczyk and Maciej Kostrzewa, independently conducted the screening. After the titles and abstracts were reviewed, the following reports were chosen for further full-text assessment. Any disagreements were resolved through discussion with all authors. The references of the reports were also screened using the snowballing procedure. Data were extracted by two authors (Monika Kowalczyk and Maciej Kostrzewa). The following data were extracted from the included studies: (1) author/s and year of publication; (2) participant characteristics (sample size, age and sex); (3) measurement tools; (4) characteristics of intervention (judo program) and a comparison of the controls or reference programs (e.g., duration, dosage, frequency); and (5) the outcomes of all analyzed variables (only statistically significant). If any data were missing, it was noted as “no data”.

### 2.4. Study Quality Assessment and Quality of Evidence

The Critical Appraisal Skills Programme (CASP) was used to assess the methodological quality of the studies [28]. CASP is commonly used in systematic reviews and is also recommended by the Cochrane Collaboration. Two authors (Monika Kowalczyk and Maciej Kostrzewa) independently reviewed each of the 22 final articles. Any discrepancies were discussed and a consensus was reached between all authors. The CASP consists of ten questions and each answer, “Yes”, “Can’t tell” and “No”, has a score of 1, 0.5 or 0, respectively. Low-quality assessment was defined as meeting 1–5 CASP checklist criteria, moderate—6–8 and high—9–10.

The Oxford level of evidence (OLE) scale was used to assess the strength of the evidence of the included studies [29]. The OLE includes 5 levels, which are presented in Table 1.

## 3. Results

### 3.1. Study Selection

**Figure 1:**
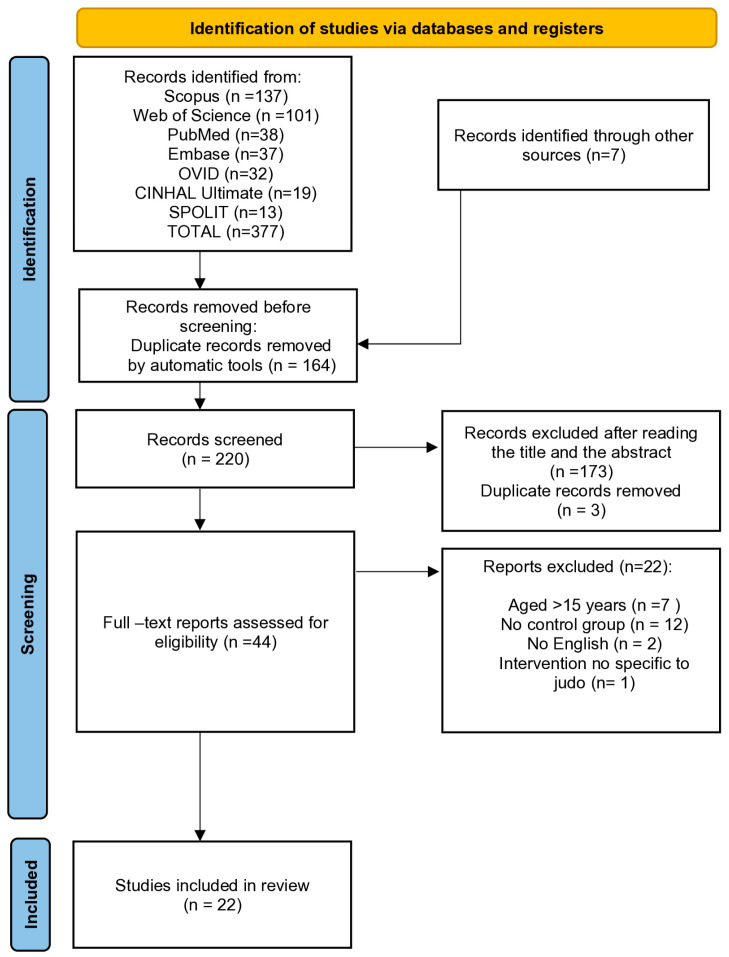
Prisma flow diagram of included studies.

A total of 377 records were identified for the study and seven were added manually, providing 384 studies for identification. After initial screening, 164 duplicates were removed and 220 studies were evaluated according to the inclusion criteria based on their titles and abstracts. Next, 44 studies qualified for a full-text assessment. Following full-text screening, 22 studies were excluded for reasons presented in the PRISMA flow diagram (Figure 1). Finally, 22 studies met the inclusion criteria and were qualified for the quality assessment.

### 3.2. The Quality of the Studies

**Figure 2:**
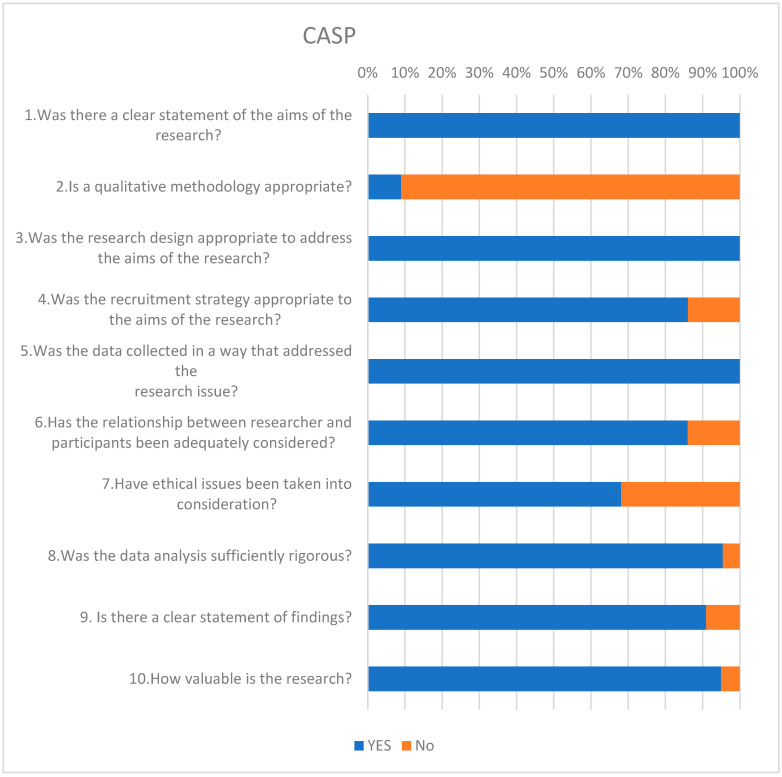
Critical Appraisal Skills Program (CASP) assessment of included studies.

All 22 studies met the inclusion criteria and were qualified for qualitative assessment according to the CASP protocol (Figure 2). The score for the included works ranged from min. 6 to max. 9 points. Studies were categorized as high-quality (n = 16) and moderate-quality (n = 6). No low-quality studies were identified. Figure 2 presents the answers to each of the ten questions based on the CASP protocol. The quality of the evidence was assessed according to the OLE: at Level 1 (L1), 1 study was a randomized controlled trial (RCT); at Level 2 (L2)—there were 5 observational studies; at Level 3 (L3), there were 16 observational studies; and at Level 4 and Level 5, there were 0 studies.

### 3.3. Characteristics of Participants

The total sample consisted of 2123 people at least five years old and at most 15 years old. The intervention judo group (JG) was represented by 792 respondents. The reference group (RG) consisted of children and adolescents practicing recreational sports such as recreational sports games (n = 49), track and field (n = 68), football (n = 100), volleyball (n = 54), karate (n = 9), and taekwondo (n = 11). The control group (CG) (n = 1039) included children and adolescents who did not engage in any additional activities, apart from PE classes as part of the school curriculum. Most (16 of 22) of the included studies involved only male participants, two covered only female participants, two examined participants of both sexes and two provided no data on the sex of the subjects. The judo intervention lasted from a minimum of three months to as long as seven years. Judo classes were held one, two or three times a week and the training time ranged from 45 to 90 min. In three studies, full data were not available on the type, intensity, and duration of the intervention. None of the authors of the studies reported any harm observed in their research.

### 3.4. Motor Competence—Overall, Locomotor and Object Control Skills

The outcomes shown in Table 2 refer to the examined motor competence (MC)—the overall, locomotor, and/or object control skills observed in JG, RG, and CG [30,31,32,33,34,35,36,37,38,39,40,41,42,43,44,45]. The authors of the 16 studies analyzed in the review used a research methodology that involved various assessment tools—MC tests and tests based on fundamental motor skills. A battery of ten motor tests was repeated in two studies only [32,33], and the authors of two other studies applied the Movement Assessment Battery for Children, Second Edition (MABC-2) [44,45]. The most important observed results are shown below.

#### 3.4.1. Judo vs. Control

The CG (not engaged in regular training) lacked significant progress compared to the JG in motor tests (jumping, running and flexibility). Only in the group of 14–15-year-olds in Drid’s et al. study did the CG record significantly higher results in hand-tapping compared to JG [31].

##### Improvement of Muscular Endurance and Strength

Sit-ups, bent-arm hang, and long jump were mostly noted as having significantly better results in the JG compared to the CG [30,31,36,37,38,40,41,42].

##### Improvement of Other Motor Competence

In five studies, the JG achieved better results in tests such as obstacles, timed runs and flexibility tests [30,31,36,38,41]. Tests such as a slalom with balls also showed better results in the JG [31,36].

#### 3.4.2. Judo vs. Other Sports

Compared to the RG (recreational sports games, football, track and field, karate, taekwondo), the JG achieved comparable or slightly better results in several motor tests (timed run, bent-arm hang, flexibility test, sit-ups, jumping sideways) [32,33,35].

#### 3.4.3. The Effect of Training Intensity and Duration

Significantly better results were noted with longer training programs (for example, lasting 3–4 years) [30,31,37]. Shorter interventions, for example, of 3.5–6 months, also brought some effects, especially in the areas of muscular endurance [38,42]. Only two interventions, lasting 3 and 8 months, respectively, showed no significant effect [43,44]. The best results were achieved with training 2–3 times per week, for a minimum of 45 to 90 min per session [32,33,37]. Only in the study by Djordjević et al. [43] was there no important outcome when judo was practiced three times a week.

### 3.5. Motor Competence—Stability

Table 3 shows a total of ten articles that investigated stability skills such as balance and postural control. Six studies analyzed dynamic or static balance [43,44,46,47,48,49], and three determined changes in posture [41,50,51], but only Walaszek et al. [39] analyzed both balance and posture. Various tests were used to measure the characteristics and only three studies repeatedly applied methods of postural evaluation such as the Moonrise method [39,50,51]. Analysis of the collected data is reflected in the following results:

#### 3.5.1. Balance Tests

##### Judo vs. Control

The outcomes of five studies showed the JG having a significant advantage in MC tests related to balance over the CG [39,46,47,48,49]. Despite the numerous benefits, some studies did not find significant differences in balance between the JG and CG, suggesting that not every study shows the clear benefits of judo training [39,43,44].

##### Judo vs. Other Sports

Only Djordjević et al. [43] investigated the balance between judo and football but both groups achieved comparable results.

#### 3.5.2. Postural Analysis

In three studies, some benefits for body posture in children practicing judo were observed [39,50,51]. In particular, the improvement of thoracic kyphosis, lumbar lordosis, and spinal rotation indicates the effect of judo on the postural alignment of the body. This may result from the characteristic movements and positions in judo, which require that a correct posture is maintained. Regular judo training results in better posture, which may be related to the physical demands of this sport, which engages the deep muscles and improves coordination and stabilization of the body.

#### 3.5.3. Long-Term Benefits

Long-lasting judo training (more than 3–4 years) gives more noticeable effects, particularly in dynamic and static balance and postural analysis [41,47]. Programs lasting longer than a year seem to influence more stability competence than short-term interventions.

## 4. Discussion

The systematic review aimed to determine the effect of judo training on the MC of children and adolescents up to 15 years old. A total of 22 studies were included in the review. Only one study was identified as an RCT study (45), but the results were not statistically significant. The intervention period in this study was only three months, which may be a crucial factor to consider when evaluating the effect of judo training on children and adolescents. Most of the studies examined were observational, with 21 in total; five rated at level 2 (L2) and 16 at level 3 (L3) on the OLE scale. Consequently, these studies contributed similarly to the overall findings. In the review, significant improvements in motor abilities were noted in all categories; overall, locomotor, object control, and stability, compared to the control groups. The most significant results concerned motor abilities such as muscular strength, endurance, speed, coordination, and flexibility. In balance and posture, a significant positive effect of judo intervention on stability competence was also noted. In comparison to other sports, the outcomes regarding MC in judo practitioners showed comparable values, and the observed differences did not indicate a clear advantage of one discipline over another.

Studies show that children and adolescents aged 6–17 who only attend physical education (PE) classes do not meet the WHO recommendations for daily (physical activity) PA [19,52,53,54]. This inactivity combined with a sedentary lifestyle leads to an increase in obesity in this age group and raises the risk of cardiovascular disease in the future [55]. In addition, children with lower motor skills are less likely to undertake any PA [4]. Participation in unorganized activities, such as playing outdoors, as well as in organized forms, provides an opportunity for more movement every day, which promotes their proper psychomotor development. However, the results of scientific studies on various forms of organized PA are not always unambiguous on this topic. Hebert et al. [52] emphasized that children participating in organized sports such as football and handball fulfilled the daily dose of PA following the global guidelines for their age. However, in sports such as gymnastics, basketball and volleyball, this relationship was not confirmed. The authors indicated the following as possible causes: a lower frequency of classes per week, sex (male subjects exercised more), and the age of the participants (the motivation to participate in PA decreased with age, which translated into time spent on PA). Similar observations were made by Rodrigues et al. [53] who conducted research among 410 children aged 6–10 participating in organized sports activities. As many as one-third of them did not meet the health-related recommendations regarding PA. Despite this, the authors noted that children’s participation in organized classes with moderate-to-vigorous exercises had a potentially positive impact on their health.

Based on the analysis of our review, it can be concluded that judo training performed by children and adolescents up to 15 years of age has a positive effect on their MC development compared to the control groups, not participating in organized, extracurricular sports activities. Similarly, Barnett et al. [56] observed that children’s participation in additional forms of PA was positively associated with MC development, particularly object control competencies, and this had a significant impact on taking up PA in adolescence [1]. Stodden et al. [56] described the relationship between PA and MC in children. The authors indicate that in early childhood, PA is important in shaping MC because in the later stage, in middle and later childhood, their fitness can be a motivating factor for being physically active in adulthood. In addition, other authors indicated that the motor development of children may be related to the amount of PA performed outside school. Opstoel et al. [57] suggested that the time spent training per week is positively related to the motor skills of those practicing at the age of 9–11. Children who trained more achieved better results in motor battery tests. Fransen et al. [58] made similar observations in the group of children aged 6–12, where motor coordination and fitness were better developed in children practicing sports for more hours a week compared to less active children [58].

Our results indicate that motor skills in children and adolescents training judo develop comparably to motor skills in those practicing other recreational sports and no significant differences were found between the groups. Pion et al. made a similar observation—they did not note any significant differences between the U13 (under 13 years old) groups practicing judo, karate, or taekwondo in the MC tests, except for the jumping sideways coordination test [35]. Importantly, only in the older age category U18 were significant differences observed between those practicing different disciplines. The results suggest that, in the older group, motor skills are better developed, training is more intensive, and the motor skills demonstrated are sport-specific for a given martial art. In another study, Opstoel et al. compared motor skills in children aged 9–11, practicing various disciplines: ball sports, dance, gymnastics, martial arts including judo, racquet sports or swimming [57]. The authors did not find significant differences in the motor skills of the participants in relation to the specificity of the practiced sport.

Judo training is one of many forms of organized classes offered to children and adolescents. Judo is a dynamic, high-intensity sport that develops strength, speed, and flexibility, as well as coordination and balance [18,20,59]. A well-formed senior judo athlete has all these MCs developed to a high level [60]. Judo training can begin at an early age, providing an opportunity to support the comprehensive motor development of those practicing from childhood. In judo, the execution of techniques is dynamic and includes various actions such as attacks (throws), counterattacks, combined techniques, and transitions from standing techniques to ground techniques (holding, elbow locks and choking) [18,19]. All these activities teach practitioners to adapt to new, rapidly changing movement situations, which has a positive effect on the body’s ability to respond to specific challenges [61]. In addition, when performing judo techniques, children must maintain balance in various positions, during both throws and defense against throws, transitioning to the ground and fighting on the ground, which promotes the development of all MCs.

In conclusion, these findings emphasize the potential benefits of judo programs aimed at enhancing the MC of children and adolescents. The regularity and length of training programs are key to achieving chronic effects. Judo is a combat sport, for which classes are conducted indoors most often in kindergartens, schools, and sports clubs as organized group classes. By participating in judo training, motor skills are developed while meeting the conditions for the health benefit dose of daily moderate-to-vigorous activity recommended by the WHO for this age group [7]. In addition to health benefits, children and adolescents comprehensively develop their MC, which is a good basis for shaping sport-specific skills and participating in competitive sports, not only in judo. The authors reported no harm in any of the studies. However, authors in previous reviews have highlighted the potential harm associated with judo training for children and adolescents (20). As a result, there is a risk, for example, that may exclude a subject from the research group. Judo is a combat sport and a contact sport, which entails a risk of injury. This information should be communicated to the parents whose children are starting judo training.

### 4.1. Limitations

The results obtained in this study must be interpreted with caution as the authors of the articles cited in this review used diverse research methodologies, which increases the risk of bias and makes their comparison difficult. In addition, individual studies lack full data on the inclusion criteria, the sex of the studied groups or a detailed description of the intervention (the duration and frequency of judo training). This prevented the authors from conducting a full meta-analysis. Additionally, the review shows that most judo participants are male. Publications often indicate that they are more engaged in PA than females, which may have an impact on the level of MC they achieve [10]. Moreover, individual studies and publications included in the analysis also had a modest number of participants in the studied group or a limited type of recreational sports in the reference groups. The main comparison group was the control group, consisting of less physically active participants, which could have affected the outcomes and their interpretations. As judo is popular among children and adolescents, it is necessary to expand research on this topic to include randomized, longitudinal studies on larger groups. A broader analysis would help to better assess the qualitative benefits of judo training, including the impact on MC development, as well as the health benefits for children and adolescents who participate in judo training.

### 4.2. Implications for Practice

Physical development of children and adolescents—judo training supports the comprehensive development of motor skills, such as strength, speed, flexibility, endurance, and coordination, making it an excellent choice for children and adolescents.

Promoting a healthy lifestyle—regular participation in judo training can help to meet the WHO recommendations regarding the level of PH, preventing overweight, obesity, and other health problems in the future.

Education in the field of movement safety—learning falls and techniques in judo reduces the risk of injuries during PA and everyday situations, improving postural stability and balance [62].

Support for psychomotor development—judo, which is a sport based on a philosophy of discipline and respect, supports not only physical development, but also shapes qualities such as self-discipline and responsibility.

Adaptation to the needs of different age groups—as the intensity and forms of judo training can be adjusted to participants’ age and level of advancement, judo can find its place in both early school education and youth programs.

Comparability with other sports—judo offers an effective alternative to other sports in terms of the comprehensive development of motor abilities, making it a valuable option in sports and educational programs in the studied age group.

## 5. Conclusions

The review found a positive effect of judo training on motor competence (MC) development in children and adolescents up to 15 years of age. Benefits were noted in the areas of muscle strength, endurance, speed, coordination and flexibility. Moreover, judo training significantly influenced the improvement of balance and body posture, suggesting that it has a positive effect on the stability skills of young judokas. Judo training brings greater benefits compared to participation in PE classes alone and is often comparable to other sports. The duration of the program and regularity of judo training are important for achieving better results in various MC tests.

## Figures and Tables

**Table 1:** Oxford level of evidence scale (OLE).

Level of Evidence	
Level 1	Meta-analysis of high-quality randomized controlled trials (RCTs) or RCTs
Level 2	Lower-quality RCTs or prospective comparative studies
Level 3	Case studies or retrospective studies
Level 4	Cases without comparison with control groups
Level 5	Case reports or expert opinions

**Table 2:** Characteristics of included studies focused on effects of judo training on motor competence—overall, locomotor and object control skills (n = 16).

Author(s) (Year)	Sample (Number, Sex)	Age (Average or Range)	Measurement Characteristics	Judo (Frequency, Dosage and/or Duration)	Reference and/or Control (Frequency, Dosage and/or Duration)	Outcomes
Sertic et al. (2009) [30]	N-87 (100% m) J—17 C—70	11 y	Ocb, Fbe, Hta, Lj, Su, Ru6, Bah	3-y program **	C: only PE classes **	J/C noted ↑ results in Ocb, Fbe, Hta, Lj, Su and Ru6.
Drid et al. (2009) [31]	N-371 (100% m) 11–12 y J1—25 C1—67 12–13 y J2—25 C2—61 13–14 y J3—35 C3—66 14–15 y J4—32 C4—60	11–15 y	Ocb, Sl, Hta, Fbe, Lj, Bah, Su, Da20	J: 2 times/w for 2 y	C: only PE classes **	J/C performed ↑ results in ages 11–15 in Ocb and Su. In J1/C1 noted ↑ results in Da20 and Lj. In J2/C2 noted ↑ results in Bah. In J3/C3 noted ↑ results in Da20, Sl and Bah. C3/J3 noted ↑ results in Hta.
Krstulovic et al. (2010a) [32]	N—79 (100% f) J—30 RSG—49	7 y	Oc, Sru, Mcs, Sar, Da20, Lj, Bt, Bah, Su, Ru3	1 session (45 min), 3 times/w for 9 mo	RSG: 1 session (45 min), 3 times/w for 9 mo	J/RSG presented ↑ results in Sru, Sar, Bah and Su.
Krstulovic et al. (2010b) [33]	N—202 (100% m) J—41 TF—68 F—38	7 y	Oc, Sru, Mcs, Sar, Da20, Lj, Bt, Bah, Su, Ru3	1 session (45 min), 3 times/w for 9 mo	TF or F: 1 session (45 min), 3 times/w for 9 mo	J/TF and J/F noted ↑ results in Bah, Su and Sar. TF/J and F/J noted ↑ results in Ru3, Bt and Da20.
Triki et al. (2012) [34]	N-96 (100% m) J—32 F—32 C—32	11 y	Sj, Cmj, Ce	6–8 h/w for min. 3 y	F: 6–8 h/w for min. 3 y C: 2 or fewer h/w of PA at school **	F/J and F/C presented ↑ results in Sj and Cmj. J/C presented ↑ results in Cmj and Ce. F/C presented ↑ results in Ce.
Pion et al. (2014) [35]	N-30 (100% m) J—10 K—9 T—11	12 y	Sar, Hg, Cmj, Sp5, Sp30, Bb, Js, Ms	No data **	No data **	J/K and T/K noted ↑ results only in Js.
Iadreev et al. (2015) [36]	N—150 (100% f) J—42 V—54 C—54	13 y	Ocb, Sl, Hta, Sar, Lj, Da20, Bah; Su	No data **	No data **	J/C and V/C in Ocb, Sl, Hta, Lj, Da20 and Su noted ↑ results.
Maśliński et al. (2015) [37]	N-66 (100% m) J—44 C—22	11–12 y	Da50, Lj, Lru, Hg, Bah, Sru, Su, Fbe	1 session (1.5 h), 3 times/w for min. 4 y	C: no formal additional PA **	J/C group had ↑ results in Lj, Bah, Sru and Fbe.
Stamenković et al. (2016) [38]	N—42 (100% m) J—22 C—20	7–8 y	Lj, Hj, Bt, Hta, Fta, Da20, Su, Kpu, Be, Fbe, Bbe, LeftSbe, RightSbe	Min. 6 mo **	C: no formal additional PA **	J/C noted ↑ results in Lj, Bt, Da20, Su, Kpu and Be.
Walaszek et al. (2017) [39]	N—24 (100% m) J—12 C—12	6 y	Vj	1 session (35–45 min), 2 times/w for 6 m	Control: no formal additional PA **	J/C noted ↑ results in take-off phases in Vj.
Missawi et al. (2018) [40]	N—119 (100% m) J—50 C—69	9–12 y	Sj, Lj	3–6 h/w for min. 2 y	C: only PE classes for 2 times to 1 h/w **	J/C performed ↑ results in Sj and Lj.
Protic-Gava et al. (2019) [41]	N—148 (100% m) J—58 C—90	12–14 y	Sar, Hj, Da20, Hta, Su, Bah, Ocb	1 h, 2 times/w for min. 3 y	C: only PE classes **	J/C had ↑ results for Sar, Hj, Bah and Ocb.
Tomac et al. (2020) [42]	N—45 (21 m) J—22 C—23	8–9 y	Su, Lj, Fbe, Hta, Ocb, Bah, Ru3	3.5 mo **	C: only PE classes **	J/C had ↑ results in Su, Lj and Ru3.
Djordjevic et al. (2021) [43]	N—84 (100% m) J—29 F—30 C—25	5–7 y	Md, Ac	1 session (1 h), 3 times/w for last 8 mo	F: 1 session (1 h), 3–4 times/w for last 8 m C: only PA according to the curriculum of the kindergarten for the last 8 mo **	J/F and J/C had no group differences. F/C noted ↑ scores for Ac and total test score.
Ludyga et al. (2021) [44]	N—42 (55% m) J—22 C—20	9–13 y	Md, Ac	1 session (1 h), 2 times/w for last 3 mo	Control: no formal additional PA **	No group differences.
Honorato et al. (2021) [45]	N—76 (100% m) J—38 C—38	12–15 y	MiHg, IeHg	3 sessions (50 min)/w for min. 1 y	C: only PE classes **	J/C noted ↑ MiHg strength in dominant and non-dominant hand and IeHg in dominant hand.

N—total sample; m—male; J—judo experimental group; C—control group; y—year/s; Ocb—obstacle course backwards; Fbe—forward bent; Hta—hand tapping, Lj—standing broad/long jump; Su—sit-ups; Ru6—6 min running; Bah—bent-arm hang; PE—physical education; **—frequency, dosage and/or duration not reported; /—in comparison to; ↑—significantly better; Sl—slalom with 3 balls, Da20—20 m dash; w—week; f—female; RSG—recreational sporting games; Oc—10m obstacle course; Sru—shuttle run; Mcs—maximal circumduction of both shoulders; Sar—sit and reach; Bt—ball throw; Ru3—3 min running; mo—month; min—minute; TF—track and field; F—football; Sj—squat jump; Cmj—counter movement jump; Ce—cycle ergometer; K—karate; T—taekwondo; Hg—handgrip; Sp5—sprint 5 m; Sp30—sprint 30 m; Bb—balancing backwards; Js—jumping sideways; Ms—moving sideways; V—volleyball; Da50—50 m dash; h—hour; PA—physical activities; Hj—high jump; Fta—feet tapping against the wall; Kpu—knee push-ups; Be—prone lying back extension; Bbe—standing backward bend; LeftSbe—standing side bend left; RightSbe—standing side bend right; Vj—vertical jump; Md—manual dexterity; Ad—aiming and catching; MiHg—maximal isometric handgrip; IeHg—isometric endurance handgrip.

**Table 3:** Characteristics of included studies focused on effects of judo training on stability motor competence (n = 10).

Author(s) (Year)	Sample (Number, Sex)	Age (Average or Range)	Measurement Characteristics	Judo (Frequency, Dosage and/or Duration)	Reference and/or Control (Frequency, Dosage and/or Duration)	Outcomes
Witkowski et al. (2014) [46]	N—51 (100% m) J—26 C—25	14–15 y	DBal, SBal	Min. 2 y	C: no formal additional PA **	J/C presented ↑ DBal.
Jankowicz-Szymańska et al. (2015) [47]	N—58 (100% m) J—29 C—29	9–13 y	SBal	1 session (1.5 h), 3 times/w for 4.5 ± 2.27 y	C: no formal additional PA **	J/C presented ↑ SBal.
Walaszek et al. (2017) [39]	N—24 (100% m) J—12 C—12	6 y	PoEv, Bal	1 session (35–45 min), 2 times/w for 6 mo	Control: no formal additional PA **	No group differences.
Walaszek et al.(2019a) [50]	N—73 (100% m) J—36 C—37	8 y	PoEv	No data **	Control: only PE classes **	J/C noted ↑ results in depth of lumbar lordosis and set of blades.
Walaszek et al. (2019b) [51]	N—74 (100% m) J—37 C—37	6 y	PoEv	1 session (45 min), 3 times/w for 6 mo	Control: only PE classes **	J/C noted ↑ results in depth of thoracic kyphosis and maximum rotation.
Protic-Gava et al. (2019) [41]	N—148 (100% m) J—58 C—90	12–14 y	PoEv	1 session (1 h), 2 times/w for min. 3 y	C: only PE classes **	J/C presented ↑ shoulder posture, chest development, abdominal wall and knee alignment.
Djordjević et al. (2021) [43]	N—84 (100% m) J—29 F—30 C—25	5–7 y	Bal	1 session (1 h), 3 times/w for last 8 mo	F: 1 session (1 h), 3–4 times/w for last 8 mo C: only PA according to the curriculum of the kindergarten for the last 8 mo **	No group differences.
Ludyga et al. (2021) [44]	N—42 (55% m) J—22 C—20	9–13 y	Bal	1 session (1 h), 2 times/w for last 3 mo	Control: no formal additional PA **	No group differences.
Barczyk-Pawelec et al. (2021) [48]	N—52 * J—26 C—26	11 y	PP, Bal	1 session (1.5 h), 3 times/w for 1 y	C: 1 session (45 min), 3 times/w of PE classes	J/C left forefoot pressure and landing noted ↑ results.
Jaworski et al. (2023) [49]	154 * 11–12 y J1—21 C1—80 13–14 y J2—19 C2—76	11–14 y	Bal	J 1: 4.3 ± 1.7 y J 2: 5.8 ± 1.3 y	C: no formal PA **	J1/C1 and J2/C2 noted ↑ values in Bal.

N—total sample; m—male; J—judo experimental group; C—control group; y—years; DBal—dynamic balance; SBal—static balance; PA—physical activities; **—frequency, dosage and/or duration, not reported; /—in comparison to; ↑—significantly better; h—hour; w—week; PoEv—postural evaluation; Bal—balance; min—minute; mo—month; PE—physical education; F—football; *—sex not reported; PP—plantar pressure.
